# Supplementary material for: The impact of nurses’ stress situation coping on somatization: a mediated moderation model
Source: PeerJ. 2024 Dec 6;12:e18658. doi: 10.7717/peerj.18658 (PMC11627075; doi:10.7717/peerj.18658)
Supplement: Supplemental Information 2 [file peerj-12-18658-s002.docx]

**English-language codebook**

**Codebook：PHQ-9 Depression scale（PHQ）**

| **Item Number** | **Item Description** | **Scoring Criteria** | **Value** | **Value Description** |
| --- | --- | --- | --- | --- |
| PHQ1 | Lack of energy or interest when doing things | None: 0  few days: 1  More than half the days: 2  Nearly every day: 3 | 0-3 | None/Mild  /Moderate  /Severe |
| PHQ2 | Feeling down, depressed, or hopeless | None: 0  few days: 1  More than half the days: 2  Nearly every day: 3 | 0-3 | None/Mild  /Moderate  /Severe |
| PHQ3 | Difficulty falling asleep, restless sleep, or sleeping too much | None: 0  few days: 1  More than half the days: 2  Nearly every day: 3 | 0-3 | None/Mild  /Moderate  /Severe |
| PHQ4 | Feeling tired or lacking in energy | None: 0  few days: 1  More than half the days: 2  Nearly every day: 3 | 0-3 | None/Mild  /Moderate  /Severe |
| PHQ5 | Loss of appetite or overeating | None: 0  few days: 1  More than half the days: 2  Nearly every day: 3 | 0-3 | None/Mild  /Moderate  /Severe |
| PHQ6 | Feeling bad about oneself or feeling like a failure, or letting oneself or one's family down | None: 0  few days: 1  More than half the days: 2  Nearly every day: 3 | 0-3 | None/Mild  /Moderate  /Severe |
| PHQ7 | Having difficulty concentrating on things, such as reading newspapers or watching TV | None: 0  few days: 1  More than half the days: 2  Nearly every day: 3 | 0-3 | None/Mild  /Moderate  /Severe |
| PHQ8 | Moving or speaking so slowly that others have noticed, or the opposite | None: 0  few days: 1  More than half the days: 2  Nearly every day: 3 | 0-3 | None/Mild  /Moderate  /Severe |
| PHQ9 | Having thoughts of being better off dead or hurting oneself in some way | None: 0  few days: 1  More than half the days: 2  Nearly every day: 3 | 0-3 | None/Mild  /Moderate  /Severe |

Scale Total Score Calculation:

Add up the scores of all items to get the total score.

Total score range: 0-27 points.

Scale Score Interpretation:

0-4 points: No depressive symptoms

5-9 points: Mild depressive symptoms

10-14 points: Moderate depressive symptoms

15-19 points: Moderately severe depressive symptoms

20-27 points: Severe depressive symptoms

**Codebook：Perceived Social Support Scale（PSSS）**

| **Item Number** | **Item Description** | **Scoring Criteria** | **Value** | **Value Description** |
| --- | --- | --- | --- | --- |
| PSSS1 | When I encounter problems, some people (such as relatives and friends) will be by my side. | Rated from 1 (strongly disagree) to 7 (strongly agree). | 1-7 | Strongly Disagree/Neutral/Strongly Agree |
| PSSS2 | I can share my joys and sorrows with some people (such as relatives and friends) | Rated from 1 (strongly disagree) to 7 (strongly agree). | 1-7 | Strongly Disagree/Neutral/Strongly Agree |
| PSSS3 | My family can provide me with tangible and concrete help | Rated from 1 (strongly disagree) to 7 (strongly agree). | 1-7 | Strongly Disagree/Neutral/Strongly Agree |
| PSSS4 | When in need, I can receive emotional help and support from my family | Rated from 1 (strongly disagree) to 7 (strongly agree). |  | Strongly Disagree/Neutral/Strongly Agree |
| PSSS5 | When I face difficulties, some people (teachers, classmates, relatives) are a true source of comfort for me | Rated from 1 (strongly disagree) to 7 (strongly agree). |  | Strongly Disagree/Neutral/Strongly Agree |
| PSSS6 | My friends are able to truly help me. | Rated from 1 (strongly disagree) to 7 (strongly agree). | 1-7 | Strongly Disagree/Neutral/Strongly Agree |
| PSSS7 | When faced with difficulties, I can rely on my friends | Rated from 1 (strongly disagree) to 7 (strongly agree). | 1-7 | Strongly Disagree/Neutral/Strongly Agree |
| PSSS8 | I can discuss my problems with my own family. | Rated from 1 (strongly disagree) to 7 (strongly agree). | 1-7 | Strongly Disagree/Neutral/Strongly Agree |
| PSSS9 | My friends can share my joys and sorrows. | Rated from 1 (strongly disagree) to 7 (strongly agree). | 1-7 | Strongly Disagree/Neutral/Strongly Agree |
| PSSS10 | In my life, there are people who care about my emotions. | Rated from 1 (strongly disagree) to 7 (strongly agree). | 1-7 | Strongly Disagree/Neutral/Strongly Agree |
| PSSS11 | My family can willingly assist me in making various decisions. | Rated from 1 (strongly disagree) to 7 (strongly agree). | 1-7 | Strongly Disagree/Neutral/Strongly Agree |
| PSSS12 | I can discuss my difficulties with my friends. | Rated from 1 (strongly disagree) to 7 (strongly agree). | 1-7 | Strongly Disagree/Neutral/Strongly Agree |

Scale Total Score Calculation:

Add up the scores of all items to get the total score.

Total score range: 12-84 points.

Scale Score Interpretation:

A low total score (12-36 points): Perceived social support is low.

A moderate total score (37-60 points): Perceived social support is moderate.

A high total score (61-84 points): Perceived social support is high.

**Codebook：Somatization Symptom Self-rating Scale (SSS)**

| **Item Number** | **Item Description** | **Scoring Criteria** | **Value** | **Value Description** |
| --- | --- | --- | --- | --- |
| SSS1 | Dizziness, Headache | Rated from 1 (None) to 4 (Severe). | 1-4 | None/Mild/Moderate/Severe |
| SSS2 | Sleep disturbances (difficulty falling asleep, vivid dreams, easy to awaken, early waking) | Rated from 1 (None) to 4 (Severe). | 1-4 | None/Mild/Moderate/Severe |
| SSS3 | Easily fatigued and weak | Rated from 1 (None) to 4 (Severe). | 1-4 | None/Mild/Moderate/Severe |
| SSS4 | Poor mood, decreased interest | Rated from 1 (None) to 4 (Severe). | 1-4 | None/Mild/Moderate/Severe |
| SSS5 | Cardiovascular symptoms (palpitations, chest tightness, chest pain, shortness of breath) | Rated from 1 (None) to 4 (Severe). | 1-4 | None/Mild/Moderate/Severe |
| SSS6 | Easily nervous, anxious, or fearful | Rated from 1 (None) to 4 (Severe). | 1-4 | None/Mild/Moderate/Severe |
| SSS7 | Prone to negative thoughts, overthinking | Rated from 1 (None) to 4 (Severe). | 1-4 | None/Mild/Moderate/Severe |
| SSS8 | Memory decline, decreased attention | Rated from 1 (None) to 4 (Severe). | 1-4 | None/Mild/Moderate/Severe |
| SSS9 | Gastrointestinal symptoms (bloating, abdominal pain, decreased appetite, constipation, diarrhea, dry mouth, nausea) | Rated from 1 (None) to 4 (Severe). | 1-4 | None/Mild/Moderate/Severe |
| SSS10 | Muscle aches (neck, shoulders, waist, back) | Rated from 1 (None) to 4 (Severe). | 1-4 | None/Mild/Moderate/Severe |
| SSS11 | Easily feel sad and cry | Rated from 1 (None) to 4 (Severe). | 1-4 | None/Mild/Moderate/Severe |
| SSS12 | Numbness, tingling, or twitching in hands, feet, or other body parts | Rated from 1 (None) to 4 (Severe). | 1-4 | None/Mild/Moderate/Severe |
| SSS13 | Blurred vision | Rated from 1 (None) to 4 (Severe). | 1-4 | None/Mild/Moderate/Severe |
| SSS14 | Easily agitated and irritable, hypersensitive to sounds | Rated from 1 (None) to 4 (Severe). | 1-4 | None/Mild/Moderate/Severe |
| SSS15 | Compulsive feelings (obsessive thoughts, compulsive behaviors) | Rated from 1 (None) to 4 (Severe). | 1-4 | None/Mild/Moderate/Severe |
| SSS16 | Limbs easily sweaty, trembling, or experiencing hot and cold flashes | Rated from 1 (None) to 4 (Severe). | 1-4 | None/Mild/Moderate/Severe |
| SSS17 | Constantly worried about falling ill | Rated from 1 (None) to 4 (Severe). | 1-4 | None/Mild/Moderate/Severe |
| SSS18 | Difficulty breathing, frequent sighing | Rated from 1 (None) to 4 (Severe). | 1-4 | None/Mild/Moderate/Severe |
| SSS19 | Discomfort in the throat, feeling of blockage | Rated from 1 (None) to 4 (Severe). | 1-4 | None/Mild/Moderate/Severe |
| SSS20 | Frequent urge to urinate | Rated from 1 (None) to 4 (Severe). | 1-4 | None/Mild/Moderate/Severe |

Scale Total Score Calculation:

Add up the scores of all items to obtain the total score.

Total score range: 20-80 points.

Scale Score Interpretation:

A low total score (20-30 points): Essentially normal psychological state, no significant emotional issues.

A moderate total score (31-38 points): May have mild emotional issues.

A high total score (39-42 points): May have moderate emotional issues.

A very high total score (43-80 points): May have severe emotional issues.

**Codebook：Coping Inventory for Stressful Situations (CISS)**

| **Item Number** | **Item Description** | **Scoring Criteria** | **Value** | **Value Description** |
| --- | --- | --- | --- | --- |
| CISS1 | I try to think about problems rationally and not let emotions take over. | Rated from 1 (Never) to 5 (Always). | 1-5 | Never/Rarely/Sometimes/Often/Always |
| CISS2 | I try to see the bright side of things. | Rated from 1 (Never) to 5 (Always). | 1-5 | Never/Rarely/Sometimes/Often/Always |
| CISS3 | I avoid thinking about this problem. | Rated from 1 (Never) to 5 (Always). | 1-5 | Never/Rarely/Sometimes/Often/Always |
| CISS4 | I try to find different ways to solve the problem. | Rated from 1 (Never) to 5 (Always). | 1-5 | Never/Rarely/Sometimes/Often/Always |
| CISS5 | I try to look at the problem from different perspectives. | Rated from 1 (Never) to 5 (Always). | 1-5 | Never/Rarely/Sometimes/Often/Always |
| CISS6 | I feel unable to cope. | Rated from 1 (Never) to 5 (Always). | 1-5 | Never/Rarely/Sometimes/Often/Always |
| CISS7 | I try to stay optimistic. | Rated from 1 (Never) to 5 (Always). | 1-5 | Never/Rarely/Sometimes/Often/Always |
| CISS8 | I feel overwhelmed by the problem. | Rated from 1 (Never) to 5 (Always). | 1-5 | Never/Rarely/Sometimes/Often/Always |
| CISS9 | I try to distract myself from thinking about this problem. | Rated from 1 (Never) to 5 (Always). | 1-5 | Never/Rarely/Sometimes/Often/Always |
| CISS10 | I analyze the problem to find possible solutions. | Rated from 1 (Never) to 5 (Always). | 1-5 | Never/Rarely/Sometimes/Often/Always |
| CISS11 | I feel powerless in this situation. | Rated from 1 (Never) to 5 (Always). | 1-5 | Never/Rarely/Sometimes/Often/Always |
| CISS12 | I try to figure out where I might have gone wrong. | Rated from 1 (Never) to 5 (Always). | 1-5 | Never/Rarely/Sometimes/Often/Always |
| CISS13 | I do my best to control my anxiety and emotions. | Rated from 1 (Never) to 5 (Always). | 1-5 | Never/Rarely/Sometimes/Often/Always |
| CISS14 | I feel crushed by the problem. | Rated from 1 (Never) to 5 (Always). | 1-5 | Never/Rarely/Sometimes/Often/Always |
| CISS15 | I try to identify the most difficult part of the problem. | Rated from 1 (Never) to 5 (Always). | 1-5 | Never/Rarely/Sometimes/Often/Always |
| CISS16 | I try to figure out what parts of the problem I can control. | Rated from 1 (Never) to 5 (Always). | 1-5 | Never/Rarely/Sometimes/Often/Always |
| CISS17 | I feel troubled by the problem. | Rated from 1 (Never) to 5 (Always). | 1-5 | Never/Rarely/Sometimes/Often/Always |
| CISS18 | I try to find something interesting or positive in the problem. | Rated from 1 (Never) to 5 (Always). | 1-5 | Never/Rarely/Sometimes/Often/Always |
| CISS19 | I seek help from friends or family to alleviate my worries. | Rated from 1 (Never) to 5 (Always). | 1-5 | Never/Rarely/Sometimes/Often/Always |
| CISS20 | I try to identify the parts of the problem that I cannot control. | Rated from 1 (Never) to 5 (Always). | 1-5 | Never/Rarely/Sometimes/Often/Always |
| CISS21 | I do my best to stay calm and not let stress affect me. | Rated from 1 (Never) to 5 (Always). | 1-5 | Never/Rarely/Sometimes/Often/Always |

Scale Dimensions:

Task-oriented Coping: Items 1, 4, 10, 12, 15, 16, 21

Emotion-oriented Coping: Items 2, 7, 11, 13, 14, 17, 18

Avoidance-oriented Coping: Items 3, 5, 6, 9, 19, 20

Scale Total Score Calculation:

Add up the scores of each dimension's items to get the total score for each dimension.

Scale Score Interpretation:

The higher the score in each dimension, the more frequently the individual uses coping strategies in that dimension.
